# Cost-effective duplex Kompetitive Allele Specific PCR markers for homologous genes facilitating wheat breeding

**DOI:** 10.1186/s12870-023-04116-y

**Published:** 2023-03-01

**Authors:** Peng Jiang, Xiangyun Fan, Guangxu Zhang, Lei Wu, Yi He, Chang Li, Xu Zhang

**Affiliations:** 1 CIMMYT-JAAS Joint Center for Wheat Diseases, Jiangsu Academy of Agricultural Sciences, Nanjing, 210014 Jiangsu China; 2 Lianyungang Institute of Agricultural Sciences, Lianyungang, 222000 Jiangsu China; 3 Collaborative Innovation Center for Modern Crop Production co-sponsored by Province and Ministry, Nanjing, 210095 Jiangsu China

**Keywords:** Favorable allele, Kompetitive Allele Specific PCR, Marker-assisted selection, Wheat breeding

## Abstract

**Background:**

Owing to successful cloning of wheat functional genes in recent years, more traits can be selected by diagnostic markers, and consequently, effective molecular markers will be powerful tools in wheat breeding programs.

**Results:**

The present study proposed a cost-effective duplex Kompetitive Allele Specific PCR (dKASP) marker system that combined multiplex PCR and KASP™ technology to yield twice the efficiency at half the cost compared with the common KASP™ markers and provide great assistance in breeding selection. Three dKASP markers for the major genes controlling plant height (*Rht-B1*/*Rht-D1*), grain hardness (*Pina-D1*/*Pinb-D1*), and high-molecular-weight glutenin subunits (*Glu-A1*/*Glu-D1*) were successfully developed and applied in approved wheat varieties growing in the middle and lower reaches of the Yangtze River and advanced lines from our breeding program. Three markers were used to test six loci with high efficiency. In the approved wheat varieties, *Rht-B1b* was the most important dwarfing allele, and the number of accessions carrying *Pinb-D1b* was much greater than that of the accessions carrying *Pina-D1b*. Moreover, the number of accessions carrying favorable alleles for weak-gluten wheat (*Null*/*Dx2*) was much greater than that of the accessions carrying favorable alleles for strong-gluten wheat (*Ax1* or Ax2^*^/*Dx5*). In the advanced lines, *Rht-B1b* and *Pinb-D1b* showed a significant increase compared with the approved varieties, and the strong-gluten (*Ax1* or Ax2^*^/*Dx5*) and weak-gluten (*Null*/*Dx2*) types also increased.

**Conclusion:**

A cost-effective dKASP marker system that combined multiplex PCR and KASP™ technology was proposed to achieve double the efficiency at half the cost compared with the common KASP™ markers. Three dKASP markers for the major genes controlling PH (*Rht-B1*/*Rht-D1*), GH (*Pina-D1*/*Pinb-D1*), and HMW-GS (*Glu-A1*/*Glu-D1*) were successfully developed, which would greatly improve the efficiency of marker-assisted selection of wheat.

**Supplementary Information:**

The online version contains supplementary material available at 10.1186/s12870-023-04116-y.

## Key message

A cost-effective duplex Kompetitive Allele Specific PCR (dKASP) marker system was proposed to achieve double efficiency at half the cost, and three dKASP markers were successfully developed and applied.

## Introduction

Numerous essential traits of wheat, including disease resistance, grain quality, and grain yield, are typical quantitative traits whose significant genotype-by-environment interactions hinder the accuracy of phenotypic evaluation [1–3]. Conversely, molecular markers reflecting differences in genome sequences are independent from environmental influences, and hence, have emerged as a powerful tool in wheat breeding programs [4, 5]. *Fhb1* to Fusarium head blight (FHB) and *Sr2* to stem rust are valuable resistance genes in wheat; however, these genes are linked in the repulsion phase. Zhang [6] successfully developed elite lines with two genes using diagnostic markers, remarkably promoting wheat resistance breeding. *Pina* and *Pinb* are the major genes controlling grain hardness, and when foreground and background selections were performed simultaneously using molecular markers, dozens of BC_2_F_2_ soft lines were obtained [7]. In recent years, many functional genes have been successfully cloned [8–10], thereby enabling the selection of more traits by diagnostic markers and ensuring the cost-efficiency of marker-assisted selection (MAS).

In the past two decades, substantial progress has been achieved in the field of molecular marker technology, which has considerably contributed to genetic research and breeding application. From hybridization-, PCR-, and to the latest sequencing-based markers [11–13], the quantity, capacity, and operability were greatly improved. Recently, single nucleotide polymorphism (SNP) markers have been receiving more attention owing to their wide distribution and excellent stability [14], and several identification technologies corresponding to different situations have also been developed, such as SNP array [15], genotyping by target sequencing (GBTS) [16], cleaved amplified polymorphic sequence (CAPS) [17], and Kompetitive Allele Specific PCR (KASP™) [11]. SNP array and GBTS, which integrate thousands of SNPs, have widely been used in genetic mapping and genetic diversity analysis; however, their high cost limited the sample size. CAPS markers were based on enzyme digestion and are suitable for a small sample. KASP™ is a homologous, fluorescence-based technology that is particularly suitable for large samples with few markers, and therefore, prefect for the MAS in large-scale breeding.

In recent years, some databases including numerous wheat KASP™ markers have been established, which will assist wheat breeders and scientists to select the most appropriate markers for MAS breeding [5, 18]. CerealsDB version 3.0 is an online resource that contains a wide range of genomic datasets for wheat [18], and provides a total of 4992 mapped KASP™ markers, which can be used in germplasm resources evaluation and MAS. Another marker database containing 122 functional KASP™ markers is proposed by Chinese Academy of Agricultural Sciences [5], and markers covering many key wheat traits including yield, disease resistance, and grain quality, have been widely used in many breeding institutions [19, 20].

KASP™ primers comprise two allele-specific primers (F1, F2) carrying fluorescence tails and one common primer (C) [11]. Generally, the two allele-specific primers correspond to a favorable and unfavorable allele. The ultimate goal of breeding selection is to obtain lines carrying favorable alleles, which enables further improvement in the cost and efficiency by optimizing the present marker system. Here a duplex KASP (dKASP) marker system was designed for certain homologous genes with the aim of providing a more efficient method for conducting MAS.

## Results

### Development of the dKASP markers

**Fig. 1:**
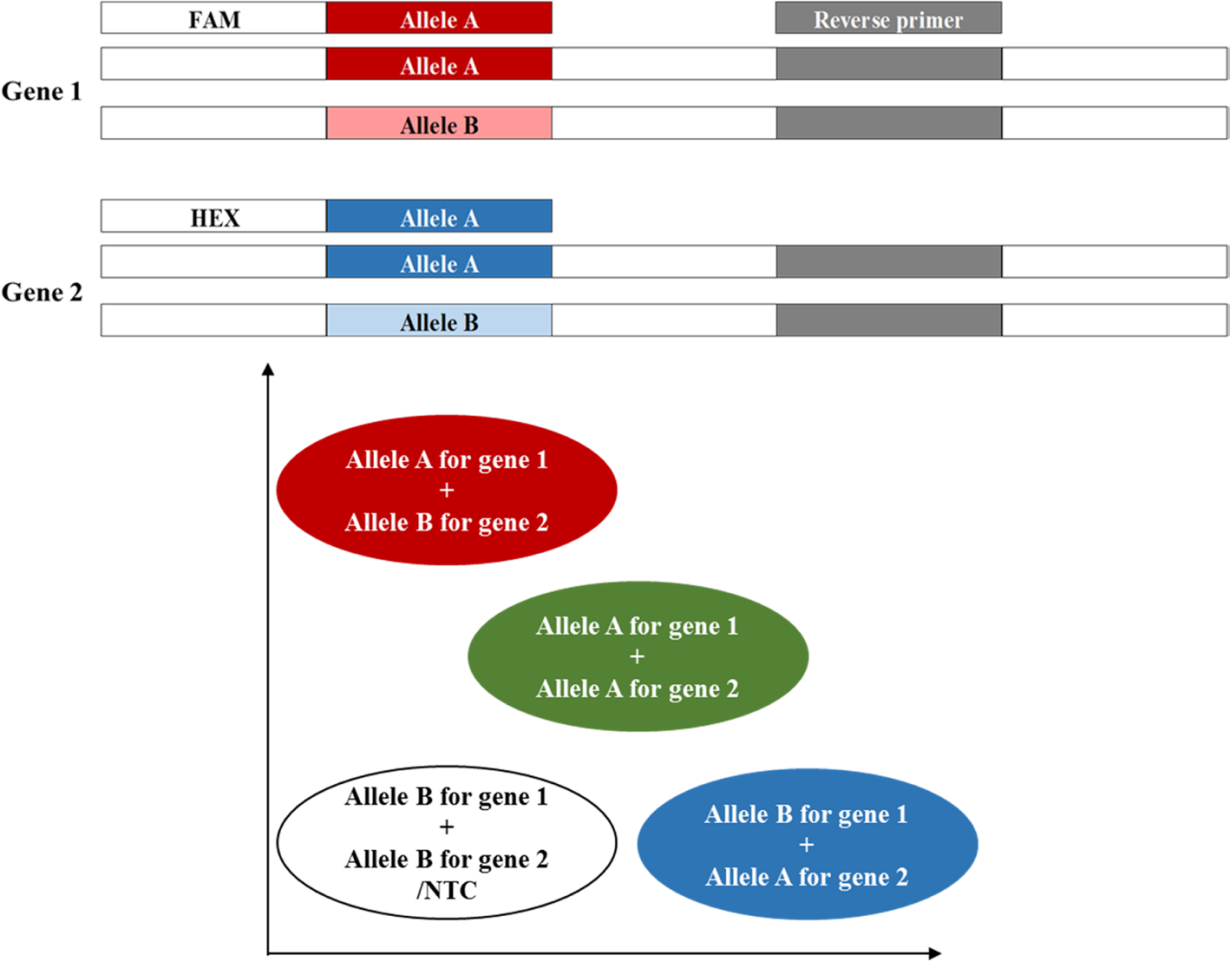
The designing schematic diagram for the primers of dKASP markers. The same color indicated the same sequence

The two allele-specific primers of the KASP™ markers usually correspond to the different alleles of one functional gene, and hence, the genotyping result included four types of alleles: AA, BB, AB, and no template control (NTC). In this study, we developed the dKASP markers whose allele-specific primers corresponded to the different alleles of the two homologous genes (Fig. 1), which also showed four types: allele A for gene 1 + allele A for gene 2, allele A for gene 1 + allele B for gene 2, allele B for gene 1 + allele A for gene 2, and allele B for gene 1 + allele B for gene 2. In this manner, two genes were identified in one reaction, and the detection efficiency was doubled while halving the cost.

**Fig. 2:**
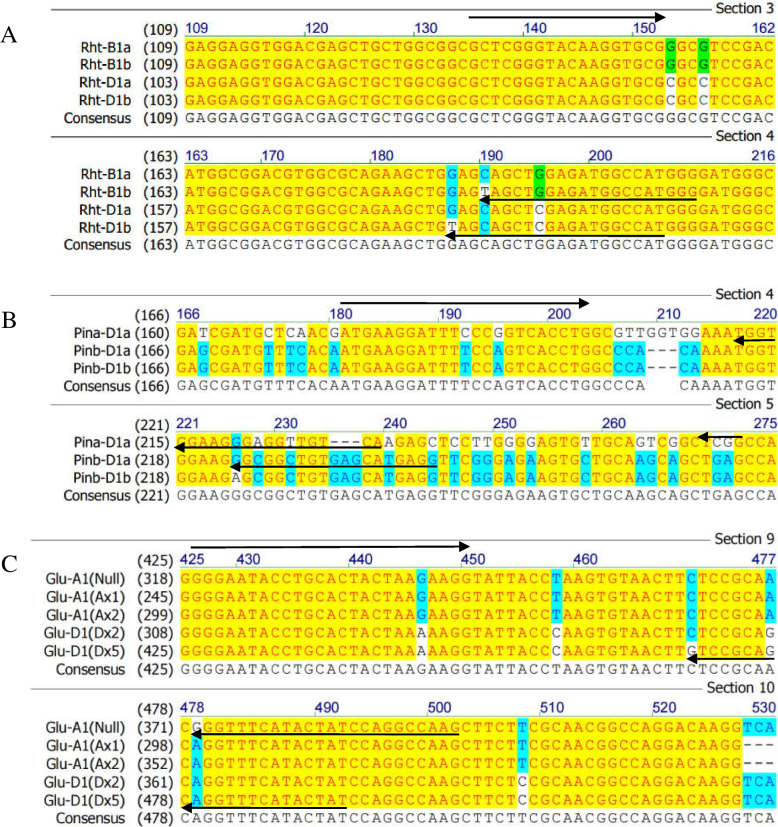
Sequence alignment of the major genes for plant height (*Rht-B1* and *Rht-D1*), grain hardness (*Pina-D1* and *Pinb-D1*), and high-molecular-weight glutenin subunits (*Glu-A1* and *Glu-D1*). **A** *Rht-B1a*, *Rht-B1b*, *Rht-D1a*, and *Rht-D1b*; **B** *Pina-D1a*, *Pinb-D1a*, and *Pinb-D1b*; **C** *Glu-A1*(*Null*), *Glu-A1*(*Ax1*), *Glu-A1*(Ax2^*^), *Glu-D1*(*Dx2*), and *Glu-D1*(*Dx5*)

**Fig. 3:**
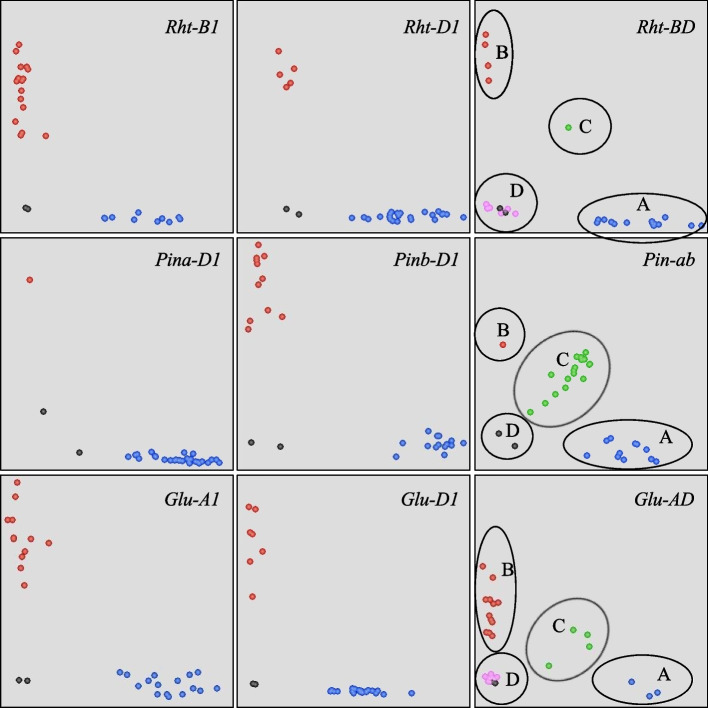
Genotyping of the common KASP™ and dKASP markers. The groups marked with A, B, C, and D corresponds to the genotypes in Table 1

In the present study, the dKASP markers for the major genes controlling plant height (PH) (*Rht-B1* and *Rht-D1*), grain hardness (GH) (*Pina-D1* and *Pinb-D1*), and high-molecular-weight glutenin subunit (HMW-GS) (*Glu-A1* and *Glu-D1*) were developed (Fig. 2 and Table 1), and the detection results of the dKASP markers were consistent with those of the diagnostic markers (some accessions were repeated twice) (Fig. 3 and Supplementary Table S1) and previous studies [19, 21–23]. *Pina-D1b* was produced by the loss of Pina, and a specific primer based on the *Pina-D1a* sequence was designed. Three allelic variations were present at *Glu-A1*, and the materials carrying *Ax1* and Ax2^*^ exhibited better gluten strength than those carrying *Null*. In the present study, *Ax1* and Ax2^*^ shared the same allele and were categorized into one group, whereas the marker, Glu-AD, was used to differentiate *Null* from *Ax1* and Ax2^*^.

**Table 1:** Sequence of the dKASP markers for the major genes controlling plant height, grain hardness, and high-molecular-weight glutenin subunits

Marker	Trait	Gene	Sequence	Genotype
Rht-BD	PH	*Rht-B1*/*Rht-D1*	F1: CCCATGGCCATCTCCAGCTA (*Rht-B1b*) F2: ATGGCCATCTCGAGCTGCTA (*Rht-D1b*) C: GCTCGGGTACAAGGTGCG	A: *Rht-B1b*/*Rht-D1a* B: *Rht-B1a*/*Rht-D1b* C: *Rht-B1b*/*Rht-D1b* D: *Rht-B1a*/*Rht-D1a*
Pin-ab	GH	*Pina-D1*/*Pinb-D1*	F1: TGACAACCTCCCTTCCACCA (*Pina-D1a*) F2: CTCATGCTCACAGCCGCC (*Pinb-D1a*) C: ATGAAGGATTTYCCRGTCACCTG	A: *Pina-D1a*/*Pinb-D1b* B: *Pina-D1b*/*Pinb-D1a* C: *Pina-D1a*/*Pinb-D1a* D: *Pina-D1b*/*Pinb-D1b*
Glu-AD	HMW-GS	*Glu-A1*/*Glu-D1*	F1: ATAGTATGAAACCTGCTGCGGAC (*Dx5*) F2: CTTGGCCTGGATAGTATGAAACCC (*Null*) C: GGGAATACCTGCACTACTAARAAGG	A: *Ax1* or Ax2^*^/*Dx5* B: *Null*/*Dx2* C: *Null*/*Dx5* D: *Ax1* or Ax2^*^/*Dx2*

### Distribution of the major genes in the approved wheat varieties in the middle and lower reaches of the Yangtze River

**Fig. 4:**
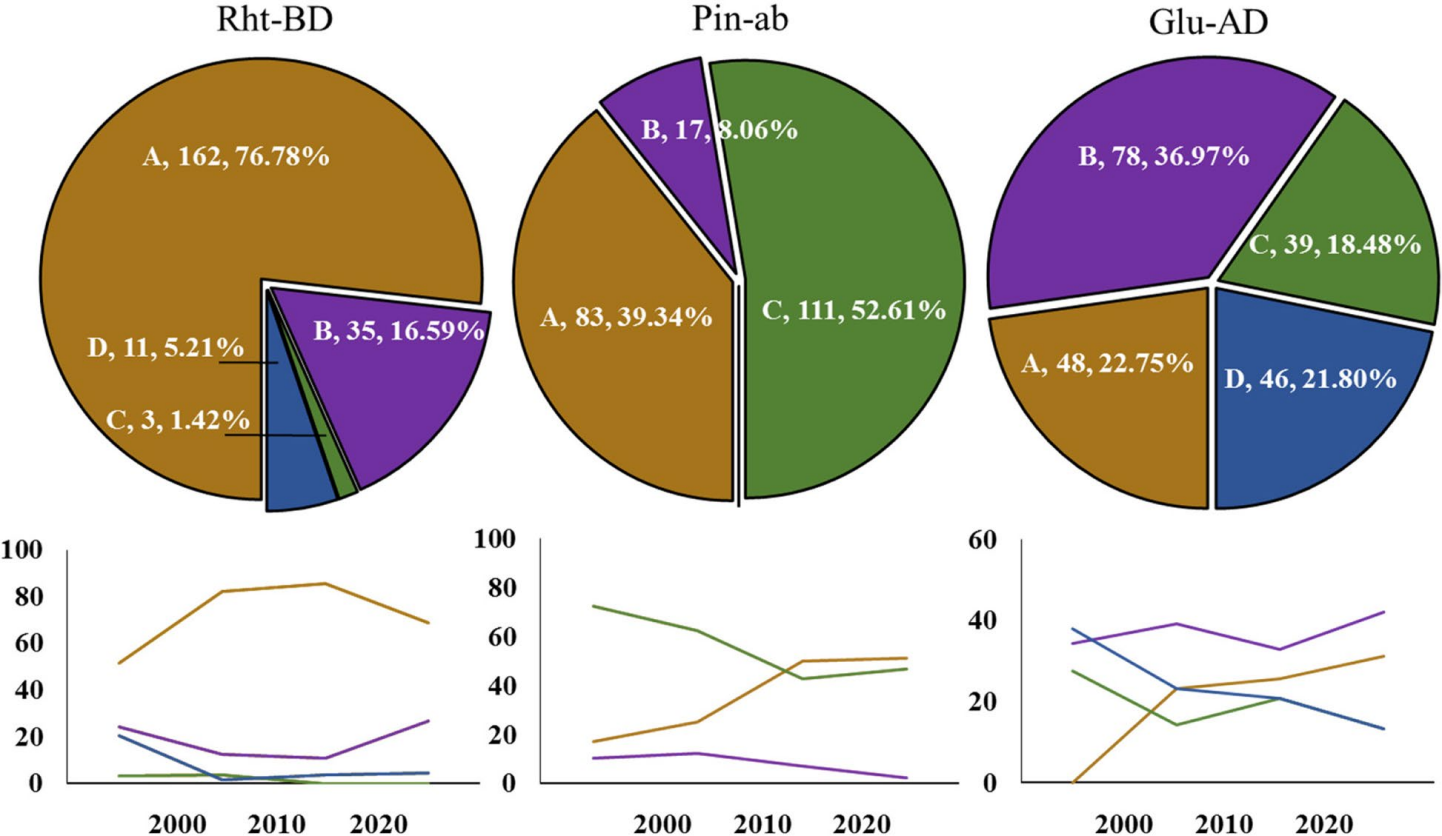
Distribution of the major genes in 212 wheat accessions and the variation tendency over time. The colors of brown, purple, green and blue indicate the genotypes of A, B, C and D in Table 1, respectively, while the numbers indicate the quantity and proportion of the corresponding genotypes

Three dKASP markers were used to screen 212 wheat accessions, including the approved varieties and elite lines, in a regional test from 1966 to 2021 (Fig. 4 and Supplementary Table S2). *Rht-B1b* was found to be the most important dwarfing allele, accounting for over three fourths of the tested accessions, and the accessions carrying *Rht-D1b* were mainly from institutions in Hubei province, while only three accessions had both *Rht-B1b* and *Rht-D1b*. Meanwhile, 11 accessions showed no dwarfing alleles at the two loci. More than half the accessions were soft type (*Pina-D1a*/*Pinb-D1a*), and the number of accessions carrying *Pinb-D1b* (39.34%) was much greater than that of the accessions carrying *Pina-D1b* (8.06%), while no *Pina-D1b*/*Pinb-D1b* type was detected. Types A (*Ax1* or Ax2^*^/*Dx5*) and B (*Null*/*Dx2*) at *Glu-A1*/*Glu-D1* were the favorable alleles for strong- and weak-gluten wheat, respectively, while the number of type B accessions was much greater than that of type A accessions, which was consistent with the classification of Chinese wheat regions based on quality [24].

More accessions carrying *Rht-B1b* were identified over time, and those without dwarfing alleles decreased significantly. Moreover, there were no accessions carrying both *Rht-B1b* and *Rht-D1b* after 2010. A substantial growth from 17.86 to 51.11% was observed for type A at *Pina-D1*/*Pinb-D1*, while an opposite trend was observed for types B and C. Both types A and B of *Glu-A1*/*Glu-D1* presented a growing trend, indicating a directional selection of wheat quality.

### Distribution of the major genes in the advanced lines

**Fig. 5:**
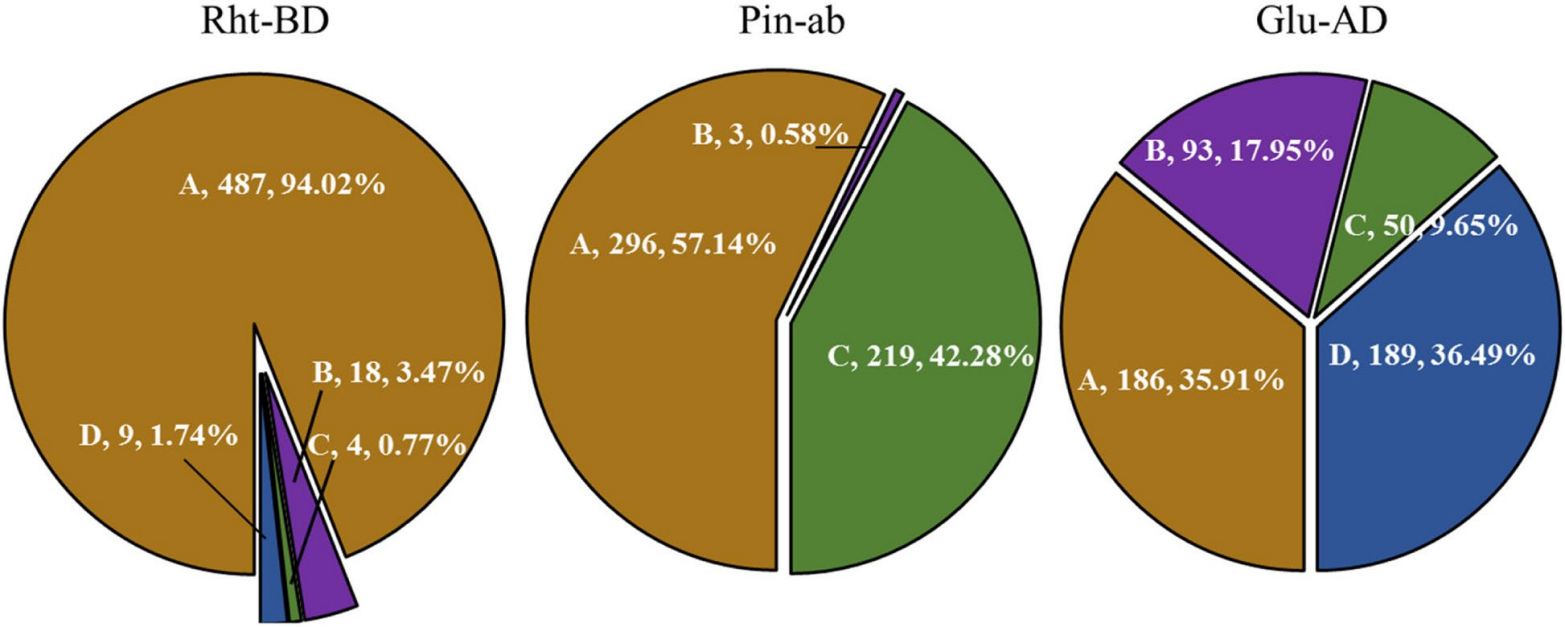
Distribution of the major genes in the advanced lines of the breeding program. The colors of brown, purple, green and blue indicate the genotypes of A, B, C and D in Table 1, respectively, while the numbers indicate the quantity and proportion of the corresponding genotypes

In total, 518 advanced lines from the breeding program were screened using the three developed dKASP markers (Fig. 5 and Supplementary Table S3). The distribution was in accordance with that of the 212 wheat accessions over time. Types B, C, and D of *Rht-B1*/*Rht-D1* accounted for only less than 10% in the advanced lines, while *Rht-B1b* was the dominant dwarfing allele. Type A of *Pina-D1*/*Pinb-D1* was the most common, while type B decreased to below 1%. The proportions of types A and D of *Glu-A1*/*Glu-D1* significantly increased compared with those in the above 212 wheat accessions.

## Discussion

According to the technique used, plant breeding can be split into four major stages [25]. Whereas the international commercial seed industries of developed countries are shifting to breeding 4.0, known as molecular design breeding, most institutions in China remain in a stage of breeding from 2.0 to 3.0, which involves selection by phenotype statistics and molecular MAS, respectively [26]. Breeding 3.0 and 4.0 involves the necessary screening of the major genes that control key traits, and hence, in these breeding techniques, a molecular marker platform plays a crucial role. Presently, electrophoretic markers, such as simple sequence repeat and sequence tagged site markers, remain the most commonly used markers in numerous breeding institutions [27]; however, their detection efficiency is low. KASP™ genotyping assay, proposed by the Laboratory of the Government Chemist in the United Kingdom, is a homogeneous fluorescence-based assay that enables the accurate bi-allelic discrimination of known SNPs and InDels. KASP™ has become popular owing to its high detection efficiency and acceptable cost [11]. In wheat breeding, numerous KASP™ markers for functional genes have been developed and widely applied [4, 5, 28]. In the present study, we proposed a cost-effective method dKASP that combines KASP™ technology and multiplex PCR to test two functional genes simultaneously to achieve twice the efficiency at half the cost.

The dKASP markers were developed based on the KASP™ technology, which involves two allele-specific primers and one common primer. Unlike the usual KASP™ markers, the two allele-specific primers of the dKASP markers are based on the sequence of two homogeneous genes. To date, many homogeneous wheat genes have been identified, here we successfully developed three dKASP markers for the major genes controlling PH (*Rht-B1*/*Rht-D1*), GH (*Pina-D1*/*Pinb-D1*), and HMW-GS (*Glu-A1*/*Glu-D1*) [29–32]. These markers were applied in the approved wheat varieties and advanced lines as well. Sequence homology limited the development of dKASP markers for numerous non-homologous genes. We attempted to apply the common multiplex PCR system involving two groups of specific primers for two non-homologous genes, such as *Fhb1*/*Fhb7* for *Fusarium* head blight (FHB) resistance and *Fhb1*/*Pm21* for resistance to FHB and Powdery mildew [8, 10, 33]. Four reaction systems with different ratios of the primers and two programs with different touchdown temperatures (61–55 °C and 65–59 °C) were tested, but unfortunately the results were not stable at present, and the system required further optimization. Other solutions need to be proposed to enhance the development of dKASP markers. Additionally, although the dKASP markers are dominant, both homozygous and heterozygous lines with favorable genotypes are usually retained to obtain more useful and noteworthy progenies during practical breeding; hence, dominant markers also engender a suitable effect. Moreover, the dKASP markers could be applied to other crops, including rice and maize, thereby improving the efficiency of MAS.

In this study, three dKASP markers were used to test six loci of the approved wheat varieties and advanced lines; this efficiently clarified the historical and present distribution of the major genes in the middle and lower reaches of the Yangtze River. Artificial selection is critical for local breeding and retains more favorable alleles, such as type A of *Rht-B1*/*Rht-D1*, type A of *Pina-D1*/*Pinb-D1*, and types A and D of *Glu-A1*/*Glu-D1*. *Rht-B1b* was the major dwarfing allele in the tested lines, which was consistent with the result of previous studies, while *Rht-D1b* was mainly distributed in the Huanghuai wheat region [34, 35]. As a serious disease in the middle and lower reaches of the Yangtze River, FHB resistance received heavy attention in local breeding, and *Rht-D1b* presented a greater negative effect on FHB resistance than *Rht-B1b* [36]. This might be due to its linkage drag with resistance QTL and its positive effects on spikelet density [35, 37]. The frequent use of elite parents, such as Yangmai 158, Ningmai 8, Ningmai 9, and Emai 12, might be another reason for the dominant proportion of *Rht-B1b* [19, 38, 39]. Moreover, decreased genetic diversity is a critical problem in modern breeding, and hence, the exploration and application of genetic resources have been receiving more attention recently. With the application of resistance genes and prevention technology to FHB, we could attempt to introduce *Rht-D1b* into the present breeding program to improve the genetic diversity and agronomic traits of wheat [35, 40].

With the development of society, wheat quality has been receiving increasing attention. GH is an important index for evaluating the quality and certifying the variety, and HMW-GS has proved to be significantly correlated with the processing quality of wheat [41, 42]. *Pina-D1a*/*Pinb-D1b* (type A) had significantly softer grain, higher break flour yield, flour yield, milling score, and loaf volume than *Pina-D1b*/*Pinb-D1a* (type B) [43, 44]. Ma et al. detected *Pina-D1*/*Pinb-D1* in 1787 accessions, and the number of accessions carrying *Pina-D1a*/*Pinb-D1b* was much greater than that of accessions carrying *Pina-D1b*/*Pinb-D1a* [45]. Similar results were observed in numerous other studies [46, 47]. In the present study, both the favorable types of strong-gluten wheat varieties, i.e., those with type A of *Pina-D1*/*Pinb-D1* and type A of *Glu-A1*/*Glu-D1*, exhibited a significant increase in the advanced lines, thereby meeting the demand for strong-gluten wheat varieties in the market. Overall, 118 lines (22.78%) with favorable types for strong-gluten wheat varieties were selected in addition to 37 lines (7.14%) with favorable types for weak-gluten wheat varieties, including those with type C of *Pina-D1*/*Pinb-D1* and type B of *Glu-A1*/*Glu-D1*. This provided abundant candidate lines for special usage wheat breeding. Meanwhile, Ax2* has rarely been identified in Chinese wheat accessions [48–50]; hence, the favorable type of strong-gluten at *Glu-A1* might mostly be *Ax1.* Therefore, the dKASP markers of Pin-ab and Glu-AD can be useful for the directional selection of wheat quality breeding.

## Conclusion

In the present study, a cost-effective dKASP marker system that combined multiplex PCR and KASP™ technology was proposed to achieve double the efficiency at half the cost compared with the common KASP™ markers. Three dKASP markers for the major genes controlling PH (*Rht-B1*/*Rht-D1*), GH (*Pina-D1*/*Pinb-D1*), and HMW-GS (*Glu-A1*/*Glu-D1*) were successfully developed and applied in the approved wheat varieties and advanced lines, and indicated a dominant proportion of *Rht-B1b* and directional selection of wheat quality in the present breeding program.

## Materials and methods

### Plant materials

This study used three panels of wheat lines. The first included 21 Chinese wheat varieties or accessions and one introduced accession that were used for marker development (Table S1). The second included 212 wheat varieties approved from 1966 to 2021 growing in the middle and lower reaches of the Yangtze River, which represented the distribution of the tested genes in modern wheat varieties (Table S2). The third included 518 F_5_ lines developed by our breeding program showing the application of favorable alleles (Table S3). The total genomic DNA of the materials was isolated from young leaf tissues using the cetyltrimethylammonium bromide (CTAB) method.

### Development and application of the dKASP markers

Three homologous gene groups were selected for marker development (Table 2). Diagnostic markers were synthesized according to Rasheed [5]. A function module of AlignX in software Vector NTI was used to search for sequence differences. Specific primers were artificially designed based on the differences, evaluated by Primer 6.0, and synthesized by Shanghai Sangon Biotech Co., Ltd. Standard FAM (5′GAAGGTGACCAAGTTCATGCT 3′) and HEX (5′ GAAGGTCGGAGTCAACGGATT 3′) tails were added to the front of the allele-specific primers F1 and F2, respectively.

**Table 2:** Information regarding homologous genes selected for marker development

Trait	Gene	Allele	Accession number
Plant height	*Rht-B1*	*Rht-B1a*	FR668586.2
		*Rht-B1b*	FN649763.1
	*Rht-D1*	*Rht-D1a*	AJ242531.1
		*Rht-D1b*	JF930281.1
Grain hardness	*Pina-D1*	*Pina-D1a*	DQ363911
		*Pina-D1b*	–
	*Pinb-D1*	*Pinb-D1a*	DQ363913
		*Pinb-D1b*	DQ363914
High-molecular-weight glutenin subunits	*Glu-A1*	*Null*	AF145590.1
		*Ax1*	X61009
		Ax2^*^	M22208.2
	*Glu-D1*	*Dx2*	BK006460.1
		*Dx5*	DQ211818.1

KASP™ assays were performed in 384-well PCR plates with 2.5 μL of KASP™ V4.0 2X Master mix standard ROX (LCG Genomics, Beverly, USA), 0.07 μL of assay mix (12 μL of each allele-specific forward primer [100 μM], 30 μL of reverse primer [100 μM], and 46 μL of TRIS [10 mM, pH 8.3]), and 2.43 μL of 30 ng μL^− 1^ genomic DNA. The PCR program was as follows: 94 °C for 15 min; 10 touchdown cycles of 94 °C for 20 s and 61 °C–55 °C for 60 s (decreasing by 0.6 °C per cycle); and 26 cycles of 94 °C for 20 s and 55 °C for 60 s. The PCR reactions were conducted in Hydrocycler 16 water instrument (LGC Genomics, Beverly, USA), and PCR fluorescence detection was performed using PHERAstar (BMG LABTECH, Germany) microplate reader. KlusterCaller software (LGC Genomics, Beverly, USA) was used for data analysis.

## Supplementary Information

**Additional file 1: Table S1.** Genotyping of the diagnostic markers and dKASP markers for plant height, grain hardness, and high-molecular-weight glutenin in 22 wheat varieties and germplasm resources. A, B, C, and D correspond to the genotypes in Table 1.

**Additional file 2: Table S2.** Genotyping of the dKASP markers for plant height, grain hardness, and high-molecular-weight glutenin in 212 wheat varieties approved from 1966 to 2021 growing in the middle and lower reaches of the Yangtze River. A, B, C, and D correspond to the genotypes in Table 1.

**Additional file 3: Table S3.** Genotyping of the dKASP markers for plant height, grain hardness, and high-molecular-weight glutenin in 518 F_5_ lines from the breeding program. A, B, C, and D correspond to the genotypes in Table 1.

## Data Availability

The data that support the findings of this study are available in the supplementary material of this article.

## Abbreviations

dKASP: Duplex Kompetitive Allele Specific PCR
FHB: *Fusarium* head blight
MAS: Marker-assisted selection
SNP: Single nucleotide polymorphism
GBTS: Genotyping by target sequencing
CAPS: Cleaved amplified polymorphic sequence
CTAB: Cetyltrimethylammonium bromide
NTC: No template control
PH: Plant height
GH: Grain hardness
HMW-GS: High-molecular-weight glutenin subunit

## References

[1] Balyan HS, Gupta PK, Kumar S, Dhariwal R, Jaiswal V, Tyagi S, Agarwal P, Gahlaut V, Kumari S (2013). Genetic improvement of grain protein content and other health-related constituents of wheat grain. Plant Breed.

[2] Nannuru VKR, Windju SS, Belova T, Dieseth JA, Alsheikh M, Dong Y, McCartney CA, Henriques MA, Buerstmayr H, Michel S (2022). Genetic architecture of *fusarium* head blight disease resistance and associated traits in Nordic spring wheat. Theor Appl Genet.

[3] Cao S, Xu D, Hanif M, Xia X, He Z (2020). Genetic architecture underpinning yield component traits in wheat. Theor Appl Genet.

[4] Su Z, Jin S, Zhang D, Bai G (2018). Development and validation of diagnostic markers for *Fhb1* region, a major QTL for *fusarium* head blight resistance in wheat. Theor Appl Genet.

[5] Rasheed A, Wen W, Gao F, Zhai S, Jin H, Liu J, Guo Q, Zhang Y, Dreisigacker S, Xia X (2016). Development and validation of KASP assays for genes underpinning key economic traits in bread wheat. Theor Appl Genet.

[6] Zhang X, Rouse MN, Nava IC, Yue J, Anderson JA (2016). Development and verification of wheat germplasm containing both *Sr2* and *Fhb1*. Mol Breed.

[7] Rai A, Mahendru-Singh A, Raghunandan K, Kumar TPJ, Sharma P, Ahlawat AK, Singh SK, Ganjewala D, Shukla RB, Sivasamy M (2019). Marker-assisted transfer of *Pina-D1a* gene to develop soft grain wheat cultivars. 3 Biotech.

[8] Su Z, Bernardo A, Tian B, Chen H, Wang S, Ma H, Cai S, Liu D, Zhang D, Li T (2019). A deletion mutation in *TaHRC* confers *Fhb1* resistance to *fusarium* head blight in wheat. Nat Genet.

[9] Chai L, Xin M, Dong C, Chen Z, Zhai H-j, Zhuang J, Cheng X, Wang N, Geng J, Wang X (2022). A natural variation in ribonuclease H-like gene underlies *Rht8* to confer “green revolution” trait in wheat. Mol Plant.

[10] He H, Zhu S, Zhao R, Jiang Z, Ji Y, Ji J, Qiu D, Li H, Bie T (2018). *Pm21*, encoding a typical CC-NBS-LRR protein, confers broad-spectrum resistance to wheat powdery mildew disease. Mol Plant.

[11] Semagn K, Babu R, Hearne S, Olsen M (2014). Single nucleotide polymorphism genotyping using Kompetitive Allele Specific PCR (KASP): overview of the technology and its application in crop improvement. Mol Breed.

[12] Chao S, Sharp PJ, Worland AJ, Warham EJ, Koebner RM, Gale MD (1989). RFLP-based genetic maps of wheat homoeologous group 7 chromosomes. Theor Appl Genet.

[13] Somers DJ, Isaac P, Edwards K (2004). A high-density microsatellite consensus map for bread wheat (*Triticum aestivum* L.). Theor Appl Genet.

[14] Liao P, Lee K (2010). From SNPs to functional polymorphism: the insight into biotechnology applications. Biochem Eng J.

[15] Wang S, Wong D, Forrest K, Allen A, Chao S, Huang B, Maccaferri M, Salvi S, Milner S, Cattivelli L (2014). Characterization of polyploid wheat genomic diversity using a high-density 90,000 single nucleotide polymorphism array. Plant Biotechnol J.

[16] Xu Y, Yang Q, Zheng H, Sang Z, Guo Z, Peng H, Zhang C, Lan H, Wang Y, Wu K (2020). Genotyping by target sequencing (GBTS) and its applications. Sci Agric Sin.

[17] Li L, Liu J, Xue X, Li C, Yang Z, Li T (2018). CAPS/dCAPS designer: a web-based high-throughput dCAPS marker design tool. Sci China Life Sci.

[18] Wilkinson PA, Winfield MO, Barker GLA, Tyrrell S, Bian X, Allen AM, Burridge A, Coghill JA, Waterfall C, Caccamo M (2016). CerealsDB 3.0: expansion of resources and data integration. BMC Bioinformatics.

[19] Jiang P, Zhang P, Yao J, Wu L, He Y, Li C, Ma H, Zhang X (2022). Phenotypic characteristics and related gene analysis of Ningmai series wheat varieties. Sci Agric Sin.

[20] Wang J, Wu X, Hu W, Zhang X, Zhang Y, Gao D, Bie T, Zhang B (2019). Kompetitive allele specific PCR( KASP) assays for functional genes of important trait in Yangmai series wheat cultivars ( lines). J Triticeae Crops.

[21] Wang H, Gao C, Zhang P, Zhang Y, Zhang P, MA H (2017). Allelic variation of puroindoline alleles in bread wheats from lower-middle reaches of the Yangtze River lower. J Triticeae Crops.

[22] Yang D, Yao JB, Yang X, Zhou M, HX MA (2015). Composition of HMW-GS in wheat germplasm from the middle and low reaches of Yangtze River. Acta Agric Boreali Sin.

[23] Zhang PP, Ma HX, Yao JB, Zhou M, Yang X, Zhang P, Yang D (2014). Subunit composition of glutenin in common wheat of Jiangsu province. Jiangsu J Agric Sci.

[24] He Z, Lin Z, Wang L, Xiao Z, Wan F, Zhuang Q (2002). Classification on Chinese wheat regions based on quality. Sci Agric Sin.

[25] Wallace J, Rodgers-Melnick E, Buckler E (2018). On the road to breeding 4.0: unraveling the good, the bad, and the boring of crop quantitative genomics. Annu Rev Genet.

[26] Zhang X, Qian Q, Zhang J, Deng X, Wan J, Xu Y (2021). Transforming and upgrading off-season breeding in Hainan through molecular plant breeding. Sci Agric Sin.

[27] Garrido-Cardenas JA, Mesa-Valle C, Manzano-Agugliaro F. Trends in plant research using molecular markers. Planta. 2018;247(3):543–57. 10.1007/s00425-017-2829-y 29243155

[28] Hewitt T, Zhang J, Huang L, Upadhyaya N, Li J, Park R, et al. Wheat leaf rust resistance gene Lr13 is a specific Ne2 allele for hybrid necrosis. Mol Plant. 2021;14(7):1025–8. 10.1016/j.molp.2021.05.010 33965633

[29] Peng J, Richards DE, Hartley NM, Murphy GP, Devos KM, Flintham JE, Beales J, Fish LJ, Worland AJ, Pelica F (1999). ‘Green revolution’ genes encode mutant gibberellin response modulators. Nature.

[30] De Bustos A, Rubio P, Jouve N (2000). Molecular characterisation of the inactive allele of the gene *Glu-A1* and the development of a set of AS-PCR markers for HMW glutenins of wheat. Theor Appl Genet.

[31] Anderson OD (2009). EST mining for structure and expression of genes in the region of the wheat high-molecular-weight glutenin loci. Genome.

[32] Simeone MC, Gedye KR, Mason-Gamer R, Gill BS, Morris CF (2006). Conserved regulatory elements identified from a comparative puroindoline gene sequence survey of *Triticum* and *Aegilops* diploid taxa. J Cereal Sci.

[33] Wang H, Sun S, Ge W, Zhao L, Hou B, Wang K, et al. Horizontal gene transfer of Fhb7 from fungus underlies fusarium head blight resistance in wheat. Science. 2020;368(6493):eaba5435. 10.1126/science.aba5435 32273397

[34] Zhang X, Yang S, Zhou Y, He Z, Xia X (2006). Distribution of the *Rht-B1b*, *Rht-D1b* and *Rht8* reduced height genes in autumn-sown Chinese wheats detected by molecular markers. Euphytica.

[35] Xu Q, Xu F, Qin D, Peng Y, Zhu Z, Dong J. Distribution of the wheat semi-dwarfing genes in China and their effects on *fusarium* head blight resistance. J Triticeae Crops. 2022;42(1):1–10.

[36] Miedaner T, Voss HH (2008). Effect of dwarfing Rht genes on *fusarium* head blight resistance in two sets of near-isogenic lines of wheat and check cultivars. Crop Sci.

[37] Srinivasachary GN, Steed A, Hollins TW, Bayles R, Jennings P, Nicholson P (2008). Semi-dwarfing *Rht-B1* and *Rht-D1* loci of wheat differ significantly in their influence on resistance to *fusarium* head blight. Theor Appl Genet.

[38] Zhang H, Zhao S, Yan Z, Huang X, Dai B, Li W (2021). Analysis of yield, quality and disease resistance traits of wheat varieties approved in Hubei province in the last two decades. J Triticeae Crops.

[39] Jiang P, Zhang X, Wu L, He Y, Zhang PP, Ma HX, Kong LR (2021). Genetic analysis for yield related traits of wheat (*Triticum aestivum* L.) based on a recombinant inbred line population from Ningmai 9 and Yangmai 158. Acta Agron Sin.

[40] Li X-P, Lan S-Q, Liu Y-P, Gale MD, Worland TJ (2006). Effects of different *Rht-B1b*, *Rht-D1b* and *Rht-B1c* dwarfing genes on agronomic characteristics in wheat. Cereal Res Commun.

[41] Li Y, Zhou R, Branlard G, Jia J (2010). Development of introgression lines with 18 alleles of glutenin subunits and evaluation of the effects of various alleles on quality related traits in wheat (*Triticum aestivum* L.). J Cereal Sci.

[42] Barro F, Rooke L, Békés F, Gras P, Tatham AS, Fido R, Lazzeri PA, Shewry PR, Barceló P (1997). Transformation of wheat with high molecular weight subunit genes results in improved functional properties. Nat Biotechnol.

[43] Martin JM, Frohberg RC, Morris CF, Talbert LE, Giroux MJ. Milling and bread baking traits associated with Puroindoline sequence type in hard red spring wheat. Crop Sci. 2001;41(1):228–34.

[44] Cane K, Spackman M, Eagles HA. Puroindoline genes and their effects on grain quality traits in southern Australian wheat cultivars. Aust J Agric Res. 2004;55(1):89–95.

[45] Ma X, Sajjad M, Wang J, Yang W, Sun J, Li X, et al. Diversity, distribution of Puroindoline genes and their effect on kernel hardness in a diverse panel of Chinese wheat germplasm. BMC Plant Biol. 2017;17:158. 10.1186/s12870-017-1101-8 PMC5607584 28931378

[46] Zhang FY, Xia XC, Dong ZD, Cui D. Distribution of puroindoline alleles in bread wheat of the yellow and Huai Valley of China and discovery of a novel puroindoline a allele without PINA protein. Mol Breed. 2012;29:371–8.

[47] Li X, Li Y, Zhang M, Xiaofen Y, Hu R, Chang J, Yang GX, Wang Y, He G (2019). Diversity of Puroindoline genes and their association with kernel hardness in Chinese wheat cultivars and landraces. Mol Breed.

[48] Yang XM, Chai SC, Li HJ, Li LH, Li X (2007). HMW-GS composition in the representative wheat landraces from China. J Triticeae Crops.

[49] Zhang X, Dong Y, You G, Wang L, Li P, Jia J. Allelic variation of *Glu-A1*,*Glu-B1* and *Glu-D1* in Chinese released wheat varieties in the last 50 years. Sci Agric Sin. 2001;34(4):355–62.

[50] Zhang X, Pang B, You G, Wang L, Jia J, Dong Y. Allelic variation and genetic diversity at *Glu-A1* loci in Chinese wheat (*Triticum aestivum* L.) germplasms. Sci Agric Sin. 2002;35(11):1302–10.

